# Effectiveness of Comfort Nursing Combined with Continuous Nursing on Patients with Colorectal Cancer Chemotherapy

**DOI:** 10.1155/2022/9647325

**Published:** 2022-06-08

**Authors:** Jing Miao, Mengting Liu, Jie Ma, Han Wang

**Affiliations:** ^1^Oncology Chemotherapy Day Ward, Peking Union Medical College Hospital, Beijing 100010, China; ^2^Pneumology and Critical Care Medicine, Peking Union Medical College Hospital, Beijing 100010, China; ^3^Cardiac Intensive Care Unit, Peking Union Medical College Hospital, Beijing 100010, China

## Abstract

**Purpose:**

To analyze the application effect of continuous nursing combined with comfort nursing on patients with colorectal cancer chemotherapy and its influence on sleep quality and immune function.

**Methods:**

The data of 96 patients with colorectal cancer in the Oncology Department of Peking Union Medical College Hospital from July 2018 to July 2020 were collected and randomized into the control group and study group according to the odd and even numbers, with 48 cases in each group. The control group received routine care during chemotherapy, and the study group implemented continuous care combined with comfort care.

**Results:**

After intervention, the results were in favor of the study group than the control group with higher compliance, higher level of various immune indicators, higher quality of life scores, and higher nursing satisfaction rate. In addition, the Generalized Anxiety Disorder (GAD-7) scores and the average Pittsburgh Sleep Quality Index (PSQI) score of the study group after intervention was drastically lower than the control group (*P* < 0.001).

**Conclusion:**

The implementation of continuous care combined with comfort care for patients with colorectal cancer undergoing chemotherapy can effectively improve sleep quality and quality of life, relieve anxiety, and yield high patient compliance, which is worthy of clinical promotion.

## 1. Introduction

Colorectal cancer is a common gastrointestinal tumor disease, which can be divided into colon cancer and rectal cancer according to different diseased sites [1, 2]. Surgery is currently the primary option for the treatment of colorectal cancer. Unfortunately, due to its hidden characters, most patients have entered the middle and advanced stages when they are diagnosed. In this regard, surgery cannot benefit patients; thus, chemotherapy is frequently used. As a damaging treatment, chemotherapy can produce strong side effects, making it prone to hair loss, abdominal pain, liver and kidney function damage [3], and further damaging the body's immune cells to undermine immune function. In addition, up to 65% of patients develop psychological stress reaction during chemotherapy, in the most direct manner of poor psychological state and sleep quality. Hence, it is essential to implement nursing intervention for patients with colorectal cancer during chemotherapy [4]. The nursing focuses on psychiatric state, nutrition support, bowel preparation for surgery, pain care, and complication care.

Continuous nursing, also known as extended nursing, is a high-quality clinical nursing service concept first proposed by the United States [5]. Its purpose is to improve patient compliance with treatment and provide patients with out-of-hospital health guidance and professional nursing. As an important part of high-quality nursing care, comfort care can organically integrate the physiology, psychology, and society of patients by providing creative and personalized nursing services for chemotherapy patients, so as to promote physical recovery and improve the quality of life in the most comfortable form [6, 7]. Traditional Chinese medicine (TCM) nursing based on “holistic concept, syndrome differentiation and treatment, meridian theory,” through skin absorption, acupoint stimulation, and meridian conduction, effectively alleviate various uncomfortable symptoms of patients with advanced cancer and has become an important content of cancer care [8, 9]. In light of these, this study explores the application effect of continuous care combined with comfort care on patients with colorectal cancer chemotherapy and its impact on sleep quality and immune function and provides more evidence for follow-up clinical care.

## 2. Materials and Methods

### 2.1. Baseline Data

The data of 96 patients with colorectal cancer in the Oncology Department of Peking Union Medical College Hospital from July 2018 to July 2020 were selected for retrospective analysis. According to the odd and even numbers of hospitalization numbers, they were divided into the study group and control group, with 48 cases in each group. The study was authorized and reviewed by hospital ethics committee (approved no. 2017-DW651).

### 2.2. Inclusion and Exclusion Criteria

Inclusion criteria were as follows: age 18–80 years old, diagnosed as colorectal cancer by pathology and treated by surgery, with normal cognitive function and audiovisual function, and the expected survival time ≥6 months.

Exclusion criteria were as follows: associated with other primary cancers, history of chemotherapy contraindications, hearing or language communication impairment, previous craniocerebral nervous system diseases such as senile dementia, craniocerebral trauma, and Parkinson's disease, and serious damage to the brain, heart, liver, kidney, and other organs.

### 2.3. Methods

#### 2.3.1. Routine Care

The control group received clinical routine care during chemotherapy, such as instructed patients to take medication on time, created a tidy and clean ward environment, strengthened daily inspection work, monitored the physical condition of patients during chemotherapy, and took preventive measures for complications.

#### 2.3.2. Continuous Care Combined with Comfort Care

The study group received clinical routine care during chemotherapy, and the specific methods were as follows.

Continuous care: (1) Established a nursing intervention team, with members including the attending physician, head nurse, and 4 nurses, with the head nurse as the team leader to carry out clinical nursing work. (2) Before enrollment, based on the clinical condition of the patients, provided psychological care and health education about bowel cancer, explained the pathogenesis and treatment process of bowel cancer, and emphasized the importance of chemotherapy to help patients understand their own diseases more comprehensively and reduced inner fears to improve his confidence in treatment and compliance with treatment [10]. (3) Strictly controlled use of analgesics of patients with severe pain while carrying out nursing care and informed patients of medication precautions. (4) Once the patient presented an abnormal condition during chemotherapy, promptly notified the doctor for corresponding treatment. Paid particular attention to the patient's physical changes during and within 1 hour after the use of chemotherapy drugs and formulated predictive nursing measures for possible complications [11]. (5) For those with sleep disorders, implemented appropriate interventions with drugs to ensure that the patients maintain adequate sleep, created a good treatment environment for the patients, and kept the ward clean and tidy.

Comfort care: (1) Recorded the patient's condition, examination results, and treatment plan in detail. Before chemotherapy, called the patients in advance to make relevant preparations and explained precautions. (2) Informed patients of the chemotherapy regimen at revisit, the progress of the disease and the duration of treatment in a timely manner based on the results of the examination. (3) After the patient is discharged from the hospital, followed up by telephone at least once a week to understand the patient's disease and guided and supervised the patient to develop a scientific diet, exercise appropriately, maintain a happy mood, and answered questions raised by the patient [12]. (4) Informed patients that they should receive chemotherapy on a regular basis, follow the doctor's advice to develop good living habits, and improved their treatment compliance and self-care ability. (5) Carry out TCM health guidance for cancer pain: according to patients' syndrome type, guide self-adjustment methods such as deep breathing, music therapy, wet hot compress, and metastatic acupoint massage.

### 2.4. Evaluation Indicators

#### 2.4.1. Treatment Compliance

The self-made chemotherapy patient compliance questionnaire by our department was used to evaluate the treatment compliance of patients after intervention. The scale includes adherence to radiotherapy, regular physical examination, scientific diet, self-protection, and disease recognition, and the results were divided into yes and no. The compliance rate was conculcated.

#### 2.4.2. Anxiety Scores

With reference to the Generalized Anxiety Disorder Scale [13] (GAD-7), the anxiety was evaluated after intervention. The scale includes 7 scoring items, each with a full score of 3 points and a total score of 21 points. The higher the score, the greater the degree of anxiety.

#### 2.4.3. Sleep Quality

With reference to the Pittsburgh Sleep Quality Index [14] (PSQI), the sleep quality of patients was evaluated after intervention. The scale includes 7 scoring items, each on a scale of 0–3, with a total score of 21 points. The higher the value, the worse the quality of sleep.

#### 2.4.4. T Cell Subsets

5 ml fasting venous blood before and after the intervention of the two groups of patients was collected and centrifuged to obtain upper serum; flow cytometry (model: Attune NxT; manufacturer: Shanghai Mojin Medical Equipment Co., Ltd.) was used to detect T cell differentiation group CD4+, CD8+, and CD4+/CD8+ ratio.

#### 2.4.5. Nursing Satisfaction

A self-made questionnaire on clinical nursing satisfaction for patients with bowel cancer chemotherapy by our department was used to evaluate the clinical satisfaction of the two groups. According to the degree of satisfaction, it was divided into very satisfied, satisfied, basically satisfied, and dissatisfied; total satisfaction = (very satisfied + satisfied + basically satisfied) number of cases/total number of cases.

#### 2.4.6. Quality of Life

The quality of life (QOL) rating scale [15] was used to evaluate the quality of life of the two groups of patients after intervention. It was evaluated from six dimensions including psychology, physiology, spirit, environment, social relations, and independence, each with a full score of 100 points; the higher the score, the higher the quality of life;

### 2.5. Statistical Methods

The data were statistically analyzed and processed by the SPSS 21.0 software, and GraphPad Prism 7 (GraphPad Software, San Diego, USA) was used to map graphics. The enumeration data were represented by (*n*%) and analyzed by the *χ*^2^ test, and the measurement data were expressed as ($\bar{x}$ ± *s*) and examined by the *t*-test. Statistical significance was accepted at *P* < 0.05.

## 3. Results

### 3.1. Comparison of Baseline Data

There was no significant difference between the two groups in gender ratio, average age, BMI value, marital status, disease type, pathological type, residence, and education level (*P* < 0.05, Table 1).

### 3.2. Comparison of Treatment Compliance

The compliance of the study group after intervention was significantly higher than that of the control group (*P* < 0.05), as given in Table 2.

### 3.3. Comparison of GAD-7 and PSQI Scores

**Figure 1:**
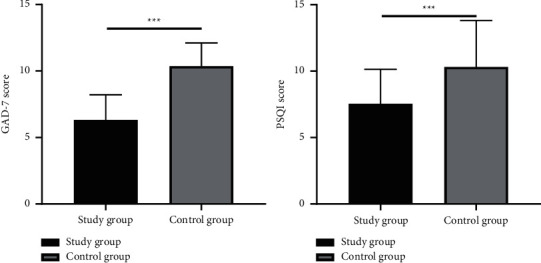
Comparison of GAD-7 scores and PSQI scores, ^*∗∗∗*^*P* < 0.001.

A drastically lower GAD-7 scores and PSQI scores of the study group after intervention than the control group were observed (*P* < 0.001), as shown in Figure 1.

### 3.4. Comparison of Various Immune Indicators

The levels of various immune indicators in the study group after intervention were observed to be considerably superior to those in the control group (*P* < 0.05), as given in Table 3.

### 3.5. Comparison of Patient Care Satisfaction

The total nursing satisfaction comparison results proved to be in favor of the study group with higher satisfaction as compared to the control group (*P* < 0.05), as given in Table 4.

### 3.6. Comparison of Quality of Life Scores

A markedly higher quality of life scores of patients in the study group after intervention was yielded in contrast to the control group (*P* < 0.001), as given in Table 5.

## 4. Discussion

With the changes in modern dietary habits, the prevalence of bowel cancer is rising, with a trend in younger population. Surgery is currently the mainstay for the treatment of the disease, yet patients in progressed stages are prone to metastasis and recurrence after surgery [16, 17]. As a result, combined adjuvant chemotherapy is urgent to improve the treatment effect and prolong the survival time of patients. Studies have found that surgery and chemotherapy can damage the immune function of patients, and the immune function is closely related to tumor recurrence and growth rate. Effective implementation is of great necessity and significance to improve the immune function, quality of life, and prognosis [18]. At present, most of the nursing work for these patients is conducted in hospitals, with the main purpose to relieve the patient's physical and mental burden, improve treatment compliance, and prevent adverse reactions that may occur during treatment. However, out-of-hospital care services are unavailable, leading to somber compliance and impeding the prognosis [19, 20].

Continuous care can realize the continuation of care from the hospital to the home, so that nursing work is no longer simply limited to the hospital, meeting the health needs of patients after discharge. Therefore, continuous care can promote the recovery of patients and plays a significant role in improving the prognosis [21, 22]. Comfortable care integrates the concept of human-centered care into the nursing process in the practice, takes the individual needs of patients as the starting point, and carries out nursing services based on the clinical conditions of the patients, effectively regulating the physical and mental conditions of the patients, keeping them in favorable condition, and consolidating the treatment effect [23]. Due to the influence by the side effects of chemotherapy and the negative emotions during cancer, the symptoms of insomnia may occur. This study showed that the average PSQI score of the study group after treatment was significantly lower than that of the control group, indicating that continuous care combined with comfort care can significantly improve the sleep quality of patients undergoing chemotherapy for colon cancer, which has been confirmed in a prior trial [24].

As is known that the lesions in patients with bowel cancer can consume nutrients in the patient's body, plus the adverse reactions caused by chemotherapy drugs, it can result in loss of appetite, weakened immunity, and high catabolism and negative nitrogen balance, compromising the chemotherapy effect [25]. This study provides continuous care for patients undergoing chemotherapy for colon cancer, develops them with scientific dietary plans, helps them establish healthy living habits, improves the body's nutritional status, and enhances immune function. And the results showed that the immune indicators of the study group after intervention are superior to the control group, suggesting that combined nursing intervention can improve the immune function of patients with colorectal cancer chemotherapy and ensure the quality of chemotherapy.

## 5. Conclusion

The implementation of continuous care combined with comfort care for patients undergoing chemotherapy for colorectal cancer can effectively improve the patient's sleep quality, enhance immune function, and produce higher satisfaction with clinical care, which is worthy of clinical promotion.

## Figures and Tables

**Table 1:** Comparison of baseline data between the two groups.

	Study group (*n* = 48)	Control group (*n* = 48)	*χ* ^2^/*t*	*P*
Gender (male/female)	26/22	25/23	0.042	0.838
Age ($\bar{x}$ ± *s*, years)	52.16 ± 4.51	52.21 ± 4.48	0.054	0.957
BMI ($\bar{x}$ ± *s*, kg/m^2^)	21.62 ± 1.05	21.58 ± 1.15	0.178	0.859
Disease type			0.300	0.584
Rectal	39 (81.25%)	41 (85.42%)		
Colon	9 (18.75%)	7 (14.58%)		
Pathological type			0.335	0.563
Adenocarcinoma	42 (87.50%)	40 (83.33%)		
Squamous carcinoma	6 (12.50%)	8 (16.67%)		
Residence			0.677	0.411
City	23 (47.92%)	19 (39.58%)		
Rural	25 (52.08%)	29 (60.42%)		

**Table 2:** Treatment compliance (*n*, %).

	Radiotherapy adherence	Physical examination	Scientific diet	Self-protection	Disease recognition
Study group (*n* = 48)	44 (91.67)	47 (97.92)	43 (89.58)	42 (87.50)	47 (97.92)
Control group (*n* = 48)	36 (75.00)	39 (81.25)	35 (72.92)	33 (68.75)	40 (83.33)
*χ* ^2^	4.800	7.144	4.376	4.937	6.008
*P*	0.028	0.008	0.036	0.026	0.014

**Table 3:** T cell subsets ($\bar{x}$ ± *s*).

Group	CD4^+^ (%)		CD8^+^ (%)		CD4^+^/CD8^+^	
	Before	After	Before	After	Before	After
Study group (*n* = 48)	32.46 ± 4.27	43.27 ± 3.28	22.81 ± 4.26	32.18 ± 3.17	1.26 ± 0.24	1.51 ± 0.35
Control group (*n* = 48)	32.52 ± 4.21	36.78 ± 3.57	22.85 ± 4.31	27.47 ± 3.26	1.31 ± 0.27	1.36 ± 0.33
*t*	0.069	9.2275	0.046	7.176	0.959	2.160
*P*	0.945	<0.001	0.964	<0.001	0.340	0.033

**Table 4:** Nursing satisfaction (*n*, %).

Group	Very satisfied	Satisfied	Generally satisfied	Unsatisfied	Total satisfaction rate
Study group (*n* = 48)	21 (43.75)	19 (39.58)	6 (12.50)	2 (4.17)	46 (95.83)
Control group (*n* = 48)	16 (33.33)	15 (31.25)	8 (16.67)	9 (18.75)	39 (81.25)
*χ* ^2^					5.031
*P*					0.025

**Table 5:** Quality of life scores ($\bar{x}$ ± *s*, points).

Group	Psychology	Physiology	Spirit	Environment	Social relation	Independence
Study group (*n* = 48)	64.38 ± 3.19	70.27 ± 3.65	68.92 ± 4.27	66.38 ± 3.28	66.26 ± 4.27	60.28 ± 4.28
Control group (*n* = 48)	58.37 ± 3.27	62.18 ± 3.28	60.27 ± 4.19	57.82 ± 3.56	59.28 ± 4.28	52.36 ± 3.76
*t*	9.115	11.422	10.018	12.252	7.999	9.632
*P*	<0.001	<0.001	<0.001	<0.001	<0.001	<0.001

## Data Availability

The datasets used during the present study are available from the corresponding author upon request.
